# Effect of lesion proximity on the regenerative response of long descending propriospinal neurons after spinal transection injury

**DOI:** 10.1186/s12868-019-0491-y

**Published:** 2019-03-18

**Authors:** Kristen Swieck, Amanda Conta-Steencken, Frank A. Middleton, Justin R. Siebert, Donna J. Osterhout, Dennis J. Stelzner

**Affiliations:** 1 Department of Cell and Developmental Biology, SUNY Upstate Medical University, 750 East Adams Street, Syracuse, NY 13210 USA; 2 Department of Neuroscience and Physiology, SUNY Upstate Medical University, 750 East Adams Street, Syracuse, NY 13210 USA; 3 Department of Biology, Slippery Rock University, 1 Morrow Way, Slippery Rock, PA 16057 USA

**Keywords:** Propriospinal neurons, Spinal cord injury, Axonal regeneration, Gene expression, Axotomy location, Intrinsic response

## Abstract

**Background:**

The spinal cord is limited in its capacity to repair after damage caused by injury or disease. However, propriospinal (PS) neurons in the spinal cord have demonstrated a propensity for axonal regeneration after spinal cord injury. They can regrow and extend axonal projections to re-establish connections across a spinal lesion. We have previously reported differential reactions of two distinct PS neuronal populations—short thoracic propriospinal (TPS) and long descending propriospinal tract (LDPT) neurons—following a low thoracic (T_10_) spinal cord injury in a rat model. Immediately after injury, TPS neurons undergo a strong initial regenerative response, defined by the upregulation of transcripts to several growth factor receptors, and growth associated proteins. Many also initiate a strong apoptotic response, leading to cell death. LDPT neurons, on the other hand, show neither a regenerative nor an apoptotic response. They show either a lowered expression or no change in genes for a variety of growth associated proteins, and these neurons survive for at least 2 months post-axotomy. There are several potential explanations for this lack of cellular response for LDPT neurons, one of which is the distance of the LDPT cell body from the T_10_ lesion. In this study, we examined the molecular response of LDPT neurons to axotomy caused by a proximal spinal cord lesion.

**Results:**

Utilizing laser capture microdissection and RNA quantification with branched DNA technology, we analyzed the change in gene expression in LDPT neurons following axotomy near their cell body. Expression patterns of 34 genes selected for their robust responses in TPS neurons were analyzed 3 days following a T_2_ spinal lesion. Our results show that after axonal injury nearer their cell bodies, there was a differential response of the same set of genes evaluated previously in TPS neurons after proximal axotomy, and LDPT neurons after distal axotomy (T_10_ spinal transection). The genetic response was much less robust than for TPS neurons after proximal axotomy, included both increased and decreased expression of certain genes, and did not suggest either a major regenerative or apoptotic response within the population of genes examined.

**Conclusions:**

The data collectively demonstrate that the location of axotomy in relation to the soma of a neuron has a major effect on its ability to mount a regenerative response. However, the data also suggest that there are endogenous differences in the LDPT and TPS neuronal populations that affect their response to axotomy. These phenotypic differences may indicate that different or multiple therapies may be needed following spinal cord injury to stimulate maximal regeneration of all PS axons.

## Background

The motor and sensory impairments that accompany injuries to the spinal cord are largely irreversible due to the inability of supraspinal neuronal populations, including the corticospinal (CST) and rubrospinal (RuST) tracts, to undergo a sustained regenerative response that can re-establish long distance connections [1, 2]. While supraspinal axons might show an initial local sprouting response immediately after injury, the expression of various inhibitory molecules in the vicinity of the lesion inhibits long distance regeneration [3]. Some functional recovery can be observed, however, if the localized axonal regrowth can interact with different populations of spinal neurons [4]. The propriospinal neuronal population, for example, has demonstrated robust regenerative and neuroplastic behaviors post-injury, which can be further enhanced using strategies such as peripheral nerve implants to create a favorable environment for repair [5–12].

Collectively, propriospinal (PS) neurons are a population of interneurons that interconnect different levels of the spinal cord. Unlike the CST and RuST neurons which originate in the cerebral cortex or brainstem and then project into the spinal gray matter, PS neurons both originate and terminate within the boundaries of the spinal cord [13]. There are several populations of propriospinal neurons; the focus of this work was the short thoracic propriospinal (TPS), and the long descending propriospinal tract neurons (LDPT). Short thoracic PS neurons arise in the thoracic spinal cord and their axons ascend or descend one or two spinal levels. This PS population has an important role in controlling postural mechanisms and axial musculature. Long descending propriospinal tract neurons (LDPT) and long ascending propriospinal neurons (LAPT) interconnect the cervical and lumbosacral enlargements. These two classes of propriospinal neurons work together with supraspinal neurons modulating and honing locomotor ability, coordination of the extremities, and postural support [14, 15].

Propriospinal neurons are increasingly attractive to the field of spinal cord injury (SCI) because the plasticity and reorganization of both spared and injured propriospinal connections can lead to functional recovery after SCI [2, 7–12]. PS axons have the ability to regenerate around incomplete spinal cord lesions and form functional neuronal circuits [7, 16]. Interestingly, even with the enhanced regenerative potential demonstrated by propriospinal axons, recent studies have demonstrated that the regenerative response of LDPT neurons and TPS neurons to a T_9_ spinal transection injury are dramatically different. TPS neurons mount a strong initial regenerative response (3 days post-axotomy), upregulating transcripts to several growth factor receptors, cell survival factors, and regeneration associated genes [17]. Additionally, TPS neurons also mount a strong apoptotic response, upregulating a handful of pro-apoptotic gene transcripts leading to cell death [17]. LDPT neurons, on the other hand, show neither a regenerative nor an apoptotic response, have a lowered expression of genes for several growth factors and their receptors, and can survive for at least 2 months post-axotomy [18, 19].

The ability of a damaged neuron to initiate and sustain regenerative activity is under the governance of different factors. While the post-injury environment is known to exert a highly inhibitory influence on the process of axonal regrowth [20–23], studies have also demonstrated that the intrinsic response of the neuron itself is another key factor [24–26]. One factor that will influence the cellular reaction to axotomy is the distance of the lesion to the neuronal cell body. Previous studies have demonstrated that a neuron will mount the strongest regenerative response if the site of axotomy is close to the cell body [5, 6, 25, 26]. If CST neurons are axotomized intracortically or spinally, there is a differential response of regeneration associated genes. While there is a significant upregulation in genes classically associated with regeneration (Atf3, Gap43, Chl1, Scg10) in the CST neurons axotomized intra-cortically, near the neuronal cell body, these were not changed in CST neurons axotomized spinally [26]. A similar effect was observed when RuST neurons (originating in the brain stem) were subjected to either a cervical or thoracic axotomy. The post-injury response of RuST neurons subjected to a cervical axotomy include upregulation of Gap43 and various tubulin proteins that were not observed after thoracic axotomy [25]. With regards to PS neurons, TPS axons travel only a few spinal segments and T_10_ injury damages their axons proximal to the TPS cell body. However, since LDPT neurons arise in the cervical and lumbar enlargements of the spinal cord, a T_10_ injury will most certainly place the site of axotomy many segments distal to their cell body. Therefore, in context with previous studies, the distance of the axotomy to the cell body may explain the differential effects observed in these two populations of PS neurons.

This study was designed to examine the effect axotomy location has on the genetic response of LDPT neurons. Specifically, we hypothesized that a proximal axotomy in LDPT neurons following a T_2_ spinal transection would result in a post-injury response in gene expression comparable to the reported changes observed in the TPS neurons following T_10_ axotomy.

## Results

The analysis in this study was focused on the response of specific genes which had significantly changed in the LDPT or TPS populations after thoracic lesions in previous studies (Table 1; 17, 18). Out of the 34 genes examined (Table 1), 28 exhibited robust and reliable expression levels above baseline in both the T_10_ injured TPS and T_2_ injured LDPT samples. Genes not surviving the initial quality control filtering with enough samples available for the first round of an analysis of variance (ANOVA) included the surface receptors Artn, Hcrt and Gfra3 and Lcn2 an immune and inflammatory gene.

**Table 1:** Specific genes selected for expression analysis

Function	Symbol	Name
Immune and/or inflammatory	Cxcl13	Chemokine (C-X-C motif) ligand 13
	Cybb	Cytochrome b-425, beta polypeptide
	Fcgr2b	Fc fragment of IgG, low affinity IIb, receptor
	Fyb	Fyn binding protein
	Itgam	Integrin alpha M
	Lgals3	Lectin, galactose binding, soluble 3
	Lcn2	Lipocalin 2
Pro/anti apoptotic	Bax	Bcl2-associated X protein
	Casp2	Caspase 2
	Casp3	Caspase 3
	Dap	Death-associated protein
	Pycard	PYD and CARD domain-containing protein
	Gadd45g	Growth arrest DNA damage inducible, gamma
Regeneration associated and/or neuroprotective	Actb	Actin, beta
	Atf3	Activating transcription factor 3
	Gadd45a	Growth arrest and DNA damage inducible 4, alpha
	Gap43	Growth-associated protein 43
	Hspb1	Heat shock protein 27
	Jun	Jun oncogene, mRNA
	Sox11	SYR-box containing gene 11
	Stmn2	Stathmin-like 2
	Stat3	Signal transducer and activator of transcription 3
	Tspo	Translocator protein
	Tubb3	Tubulin—beta 3
Surface receptor and/or growth factor	Artn	Artemin
	Bdnf	Brain-derived neurotrophic factor
	Gfra1	Glial cell line derived neurotrophic factor family receptor alpha 1
	Gfra3	Glial cell line derived neurotrophic factor family receptor alpha 3
	Hcrt	Orexin (Hypocretin)
	Igf1	Insulin-like growth factor 1
	Lifr	Leukemia inhibitory factor receptor
	Ncam1	Neural cell adhesion molecule 1
	Ntrk2	Neurotrophic tyrosine kinase, receptor type 2
	Ret	Ret proto-oncogene

Genes selected for analysis. 34 different genes were specifically chosen to be profiled at 3-days post injury. The genes chosen for analysis were previously found to be significantly up or down-regulated 3-days post injury in LDPT and/or TPS neurons following gene expression and qRT PCR array analyses [17, 18]. Expression levels of these 34 specific genes were assessed using a custom-designed multiplex  assay. Stmn2 is also known as SCG10

Further evaluation of the expression data for LDPT neurons revealed that for the genes Cybb and Sox11, only two of the four uninjured LDPT control samples generated expression values, thus rendering any kind of statistical analysis problematic. Therefore, both Cybb and Sox11 were removed from further analysis with regards to the LDPT populations. Additionally, the expression changes for another regeneration associated and neuroprotective gene, Atf3, could not be deemed statistically significant because of the high level of variability that was observed among the uninjured LDPT control group. However, the data are showing a clear trend towards a robust upregulation in Atf3 expression in LDPT neurons receiving a localized axotomy, so this was included in the analysis.

Interestingly, Bax, a pro/anti apoptotic gene and Cxcl13, an immune and inflammatory gene, also did not survive the initial filtering of data prior to the ANOVA analysis. However, in this case, it was because the expression of both genes was only observed in the TPS population. This surprising finding suggests that there are strong phenotypic differences between the LDPT and TPS neuronal populations. Also of interest was the finding that TATA box-binding protein gene (Tbp), one of two housekeeping and control genes (Hprt and Tbp), exhibited a change in expression after a lesion, and was therefore not used as a reference gene; instead it is being reported among those tested with ANOVA. These data resulted in a total of 27 genes being further analyzed in this study.

The 27 remaining predetermined genes in this study were segregated into four general functional families: pro/anti apoptotic, immune and inflammatory, regeneration associated and neuroprotective, and cell surface and growth factors. Tbp regulates gene expression by binding to the TATA box upstream of various eukaryotic genes and promotes expression [27, 28]. It could technically fit into any of the four identified families, but it is considered as being in a separate functional family of gene expression regulators.

## Response of thoracic propriospinal neurons following axotomy

Our previous study demonstrated that after spinal injury, TPS neurons initiate a robust change in expression of many different genes involved in the four functional families [17]. The current study, in part, replicated the previous work, examining the intrinsic genetic response of TPS neurons to a T_10_ level lesion. Evaluation of the response of TPS neurons to a cervical injury was not done in this study, because the previous study demonstrated there was no effect on gene expression [17]. This analysis was performed using custom-designed magnetic bead-based Luminex assays (QuantiGene Plex 2.0; Affymetrix). This technology is similar in sensitivity as qRT-PCR, which was utilized in our previous study, but affords the ability to simultaneously measure up to 36 genes in a single well, thus significantly reducing overall variance.

**Fig. 1:**
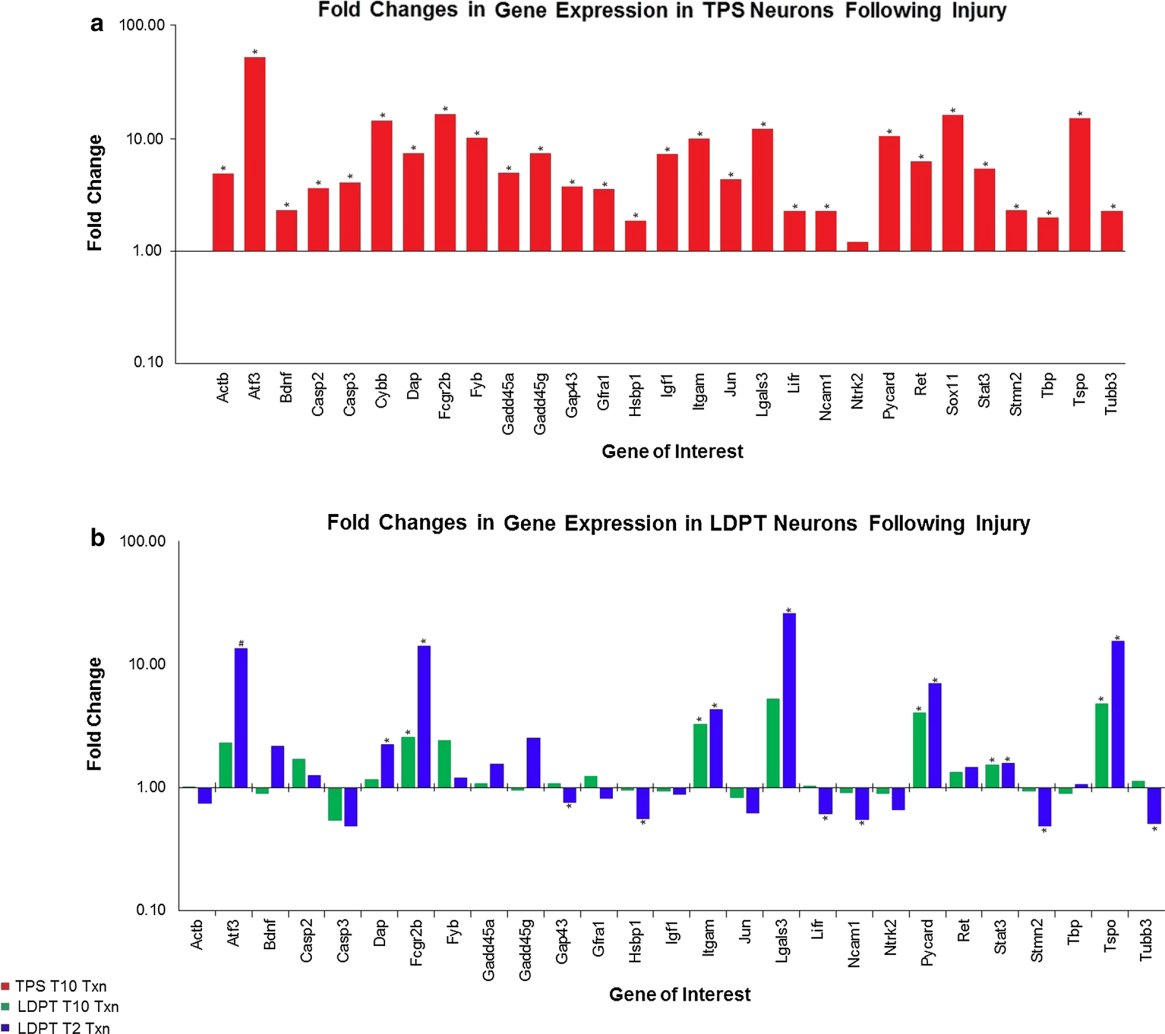
Fold changes in gene expression post spinal cord injury. The fold changes in gene expression, following spinal cord injury,  were determined for both the short Thoracic Propriospinal neurons (TPS) receiving an injury at the spinal level T_10_ (**a**), or the Long Descending Propriospinal Neurons (LDPT) that received a distal injury at T_10_ or a proximal injury at T_2_ (**b**). All changes in expression were determined by comparing the injured group to the uninjured control. Genes exhibiting a statistically significant fold change in expression compared to the uninjured control (corrected *p* value ≤ 0.10) 3 days post-injury are indicated by an asterisk (*), Gene trending towards significance but sample expression variability among the control group prevents statistical significance (#)

As expected, the expression levels of all genes examined was higher in the T_10_ injured animals compared to the controls (Fig. 1a). The magnitude of the changes in gene expression varied, ranging from a robust change (52.4 fold increase over control) for the regeneration associated gene Atf3, to a milder change, (1.2 fold increase over control) for the cell surface receptor/growth factor gene, Ntrk2. Statistically, all changes in expression were found to be significant, with the exception of Ntrk2 (*p* = 0.3801) and were observed in genes from all four functional families. These data replicate the findings in our previous work, demonstrating that TPS neurons mount a robust post-injury response [17], and validates the sensitivity and utility of multiplex bead-based technology for examining changes in gene expression.

## Response of long descending propriospinal neurons following distant axotomy

The response of LDPT neurons, with axons spanning a distance from the cervical enlargement to the lumbosacral enlargement, was characterized following a spinal transection injury at the T_10_ thoracic level. Previous work demonstrated that LDPT neurons remain relatively quiescent, or even downregulate certain genes in response to a T_10_ transection injury, a response very different from the TPS neurons [18]. In this study, following a T_10_ axotomy, the post-injury change in gene expression was again flat (Fig. 1b), with only a few genes showing increases. Moreover, 10 genes displayed a *decrease* in gene expression: Bdnf, Casp3, Gadd45g, Hspb1, Igf1, Jun, Ncam1, Ntrk2, Stmn2, and Tbp. When the overall fold changes and statistical significance were considered (Fig. 1b), LDPT neurons exhibited a much smaller response in gene expression. Moreover, only five genes examined exhibited a statistically significant (corrected *p* value < 0.10) fold change in expression: Fcgr2b and Itgam, both immune and inflammatory genes; Pycard, a pro/anti apoptotic gene; Stat3, and Tspo both regeneration associated genes. The observed LDPT response to a T_10_ injury, which is located far from the LDPT neuronal cell bodies, is quite different than the observed TPS response to the same T_10_ level injury. The findings from this study validate and further confirm the findings from our previous studies [17, 18]. It also suggests that lesion proximity might have a role in stimulating changes in gene expression.

## Response of long descending propriospinal neurons following local axotomy

The response of LDPT neurons to a local axotomy was examined to determine if a local lesion would elicit a strong regenerative response, similar to what was observed in the TPS neurons. LDPT neurons were axotomized at the spinal level T_2_, placing the site of axotomy approximately three to five spinal segments away from the cell bodies of the LDPT neurons. This is comparable to the experimental conditions of the previous analysis of TPS neurons, which are located at the T_7_ level, with an injury occurring at the T_10_ spinal level.

After a T_2_ axotomy, the response of the LDPT neurons was very different in both the levels and the direction of gene expression following injury (Fig. 1b and Table 2). Of the genes of interest, eight exhibited a divergent genomic response. Three of those genes were regeneration associated and neuroprotective genes: Actb, Gap43, Tubb3, which all were *down regulated* in LDPT neurons close to the axotomy, whereas after a distant axotomy, the expression of these genes essentially remained unchanged or increased.  The surface receptors genes, Gfra1 and Lifr were also down regulated when LDPT neurons were locally axotomized, compared to the upregulation observed after a distal axotomy. In contrast, the opposite pattern of changes was observed for the genes Bdnf, and Gadd45g, associated with antiapoptotic functions in cells. Both Bdnf and Gadd45g were *upregulated* in locally injured LDPT neurons, whereas their expression slightly declined following a distant axotomy. The final gene exhibiting a divergent change in the LDPT response to injury was the housekeeping gene candidate Tbp.

**Table 2:** Significant changes in gene expression in LDPT neurons

Gene	Observed fold change in expression		Corrected *P* values		Functional grouping
	LDPT T10 lesion	LDPT T2 lesion	LDPT T10 lesion	LDPT T2 lesion	
Dap	1.15	2.22	0.708853	*0.00365*	Pro and anti apoptotic
Fcgr2b	2.57	14.13	0.097471	*0.00027*	Immune and inflammation
Gap43	1.07	0.76	0.635246	*0.024751*	Regeneration associated and neuroprotective
Hsbp1	0.95	0.55	0.685646	*0.002492*	Regeneration associated and neuroprotective
Itgam	3.26	4.32	0.054079	*0.00187*	Immune and inflammation
Lgals3	5.26	26.75	0.362824	*0.002079*	Immune and inflammation
Lifr	1.02	0.61	0.903837	*0.010078*	Surface receptor and growth factor
Ncam1	0.91	0.54	0.650819	*0.013782*	Surface receptor and growth factor
Pycard	4.02	7.13	*0.030144*	*0.003767*	Pro and anti apoptotic
Stat3	1.53	1.57	0.066473	0.063692	Regeneration associated and neuroprotective
Stmn2	0.93	0.48	0.743167	*0.030081*	Regeneration associated and neuroprotective
Tspo	4.75	15.33	*0.04949*	*0.001578*	Regeneration associated and neuroprotective
Tubb3	1.11	0.51	0.620656	*0.015431*	Regeneration associated and neuroprotective

Significant post-hoc* p* values are shown in italics

Significant changes in gene expression in LDPT neurons. Changes in expression in LDPT neurons, depending on the location of the lesion were quantified. Five genes (Fcgr2, Itgam, Pycard, Stat3, Tspo) all demonstrated a significant increase in expression following a distal lesion at T_10_. However, when LDPT neurons were axotomized proximally at T_2_, 13 genes exhibited a significant change in expression: six were down-regulated (Gap43, Hsbp1, Lifr, Ncam1, Stmn2, Tubb3) while seven (Dap, Fcgr2b, Itgam, Lgals3, Pycard, Stat3, Tspo) were upregulated. This direct comparison strongly indicates that LDPT neurons exhibit a more dynamic intracellular response when the axotomy is located nearer the LDPT cell body

There were significant fold *increases* (corrected *p* value < 0.10; Table 2) in the expression of seven genes, including the immune and inflammatory genes Fcgr2b, Itgam, and Lgals3; the regenerative associated genes Tspo and Stat3, and the pro/anti apoptotic genes Dap and Pycard. The regenerative associated gene Atf3, trended towards significance, however the high degree of variability in the sample prevented any statistical significance. Curiously, there were significant (corrected *p* value < 0.10; Table 2) fold *decreases* in regeneration associated genes: Gap43, Hspb1, Stmn2, Tubb3, and growth factor & surface receptor genes: Lifr and Ncam1.

Notable, but non-significant changes in the expression included increases in the immune gene Fyb, the growth factor and surface receptor genes Bdnf, and Ret, of the pro/anti-apoptotic genes Casp2, and Gadd45g, and the regeneration associated and neuroprotective gene Gadd45a. Non-significant decreases in expression were observed for the growth factor and surface receptor gene Gfra1, and Ntrk2, the pro/anti apoptotic gene Casp3, and the regeneration associated and neuroprotective genes: Actb, and Jun.

Collectively, when considering the overall changes in gene expression (see Fig. 1b), the number of genes exhibiting a significant change in expression after a local injury increased almost threefold, as compared to a distant injury. Only  five genes were affected after a distant injury, while the local axotomy stimulated significant changes in the expression of  13 genes. Of these, seven demonstrated statistically significant (corrected *p* values < 0.002; Table 2) *increases* in expression. These included Dap, Pycard, Fcgr2b, Itgam, Lgals3, Stat3 and Tspo. The six remaining genes exhibited significant (corrected *p* values < 0.010; Table 2) fold *decreases*, and included Gap43, Hsbp1, Stmn2, Tubb3, Lifr and Ncam1.

**Fig. 2:**
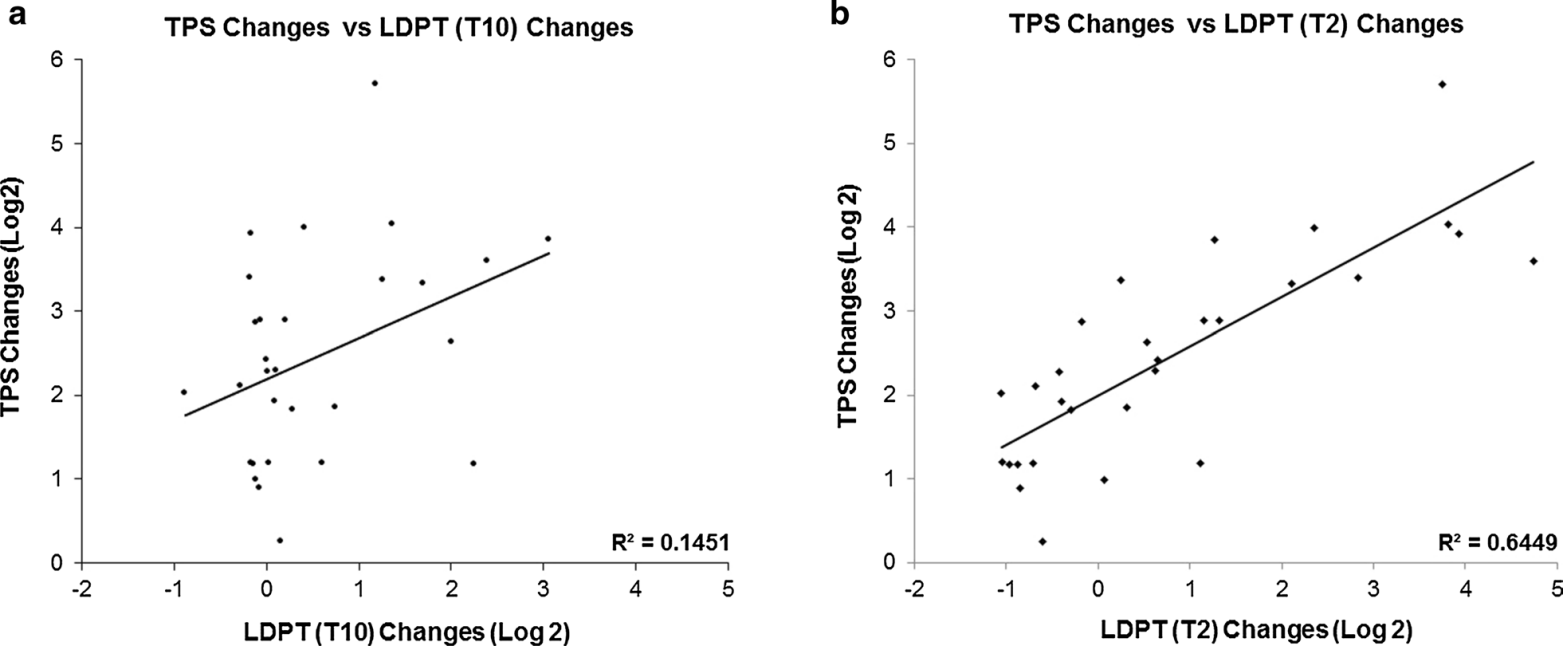
Correlation analysis of lesion distance and change in gene expression. The location of axotomy relative to the neuronal cell body is an important determinant for the post-injury responses exhibited in terms of changes in gene expression. This relationship was examined using a correlation analysis comparing the TPS neurons response to a T_10_ injury, to the response of LDPT neurons receiving an injury at T_10_ (**a**), and the response of LDPT neurons receiving an injury at T_2_ (**b**). When changes in gene expression were compared between LDPT neurons receiving a distant axotomy (T_10_) and the TPS neurons close to the injury site, the correlation was r = 0.38 (**a**). However, when the LDPT neurons near a local axotomy (T_2_) were compared to the TPS neurons, a correlation of r = 0.803 was observed (**b**)

These data clearly demonstrate that the location of axotomy relative to the neuronal cell body is an important determinant for the changes elicited in gene expression. This relationship can be further confirmed using a correlation analysis. When changes in gene expression were compared between LDPT neurons receiving a distant axotomy (T_10_) and the TPS neurons close to the injury site, the correlation was weak (Pearson r = 0.38, *p* value = 0.042) (Fig. 2a). However, when the expression changes in LDPT neurons receiving a local axotomy (T_2_) were compared to the TPS neurons, a very strong correlation was found (Pearson r = 0.803, *p* value < 0.00001). There was a much more obvious correlation between the gene expression changes in TPS and LDPT neurons receiving a local axotomy (Fig. 2b).

## Differential response of gene families

**Fig. 3:**
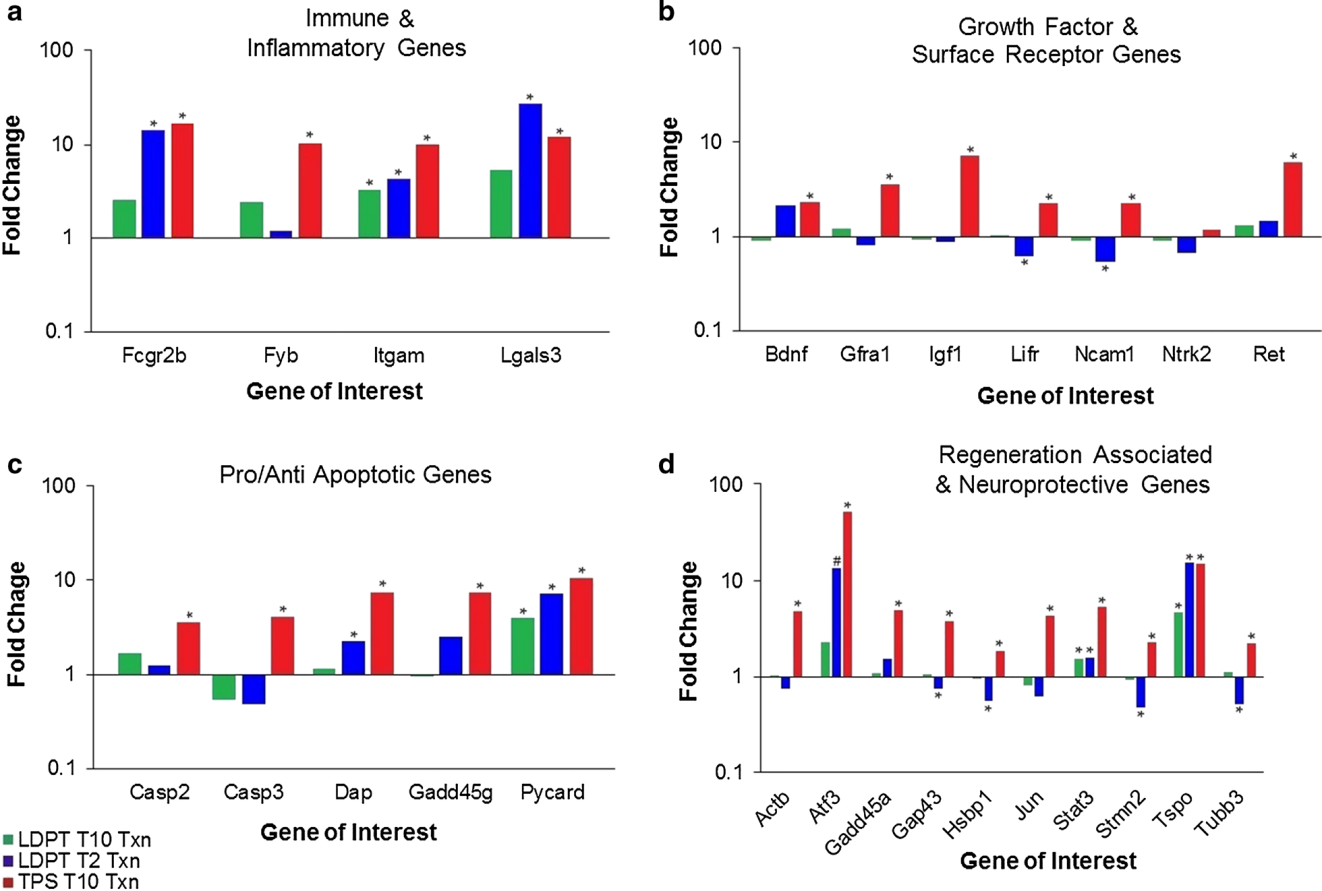
Fold changes in gene expression post spinal cord injury group by functional family. While fold changes in gene expression were already calculated and presented, genes were grouped together by their predetermined functional family (see Table 1) for better visualization of which family exhibited the most dynamic change post-injury. Genes encoding factors known to be involved with the immune and inflammatory process are grouped together (**a**), Genes encoding known growth factors and surface receptors are grouped together (**b**), Genes representing genes that are known to be pro or anti apoptotic are grouped together (**c**), and those genes that encode known neuroprotective or regeneration associated genes are grouped together (**d**). As can be seen in **a**–**d**, TPS neurons exhibited significant upregulation of genes across all four functional groupings. LDPT neurons receiving a T_2_ lesion exhibited a significant upregulation in three of the four genes (Fcgr2b, Itgam, and Lgals3) in the immune and inflammatory family (**a**) and two of the  five genes (Dap and Pycard) in the pro/anti apoptotic family (**c**), whereas in the growth factor and surface receptor family, two of the seven genes (Lifr and Ncam1) exhibited a significant downregulation in expression (**b**). In regards to the regeneration associated and neuroprotective genes family (**d**), of the 10 genes examined, one gene (Tspo) was significantly upregulated, one gene showed a trend towards significance (Atf3), and four genes (Gap43, Hspb1, Stmn2, and Tubb3) demonstrated a significant downregulation. LDPT neurons receiving a T_10_ level injury, were relatively quiescent, with significant changes in expression being found for only four of the total genes. This clustering of genes by family shows the most dynamic response in LDPT neurons receiving a T_2_ injury were among the genes involved with inflammation and the immune response, followed by the genes associated with neuroprotection and regeneration. Genes exhibiting a statistically significant fold change in expression compared to the uninjured control (corrected *p* value ≤ 0.10) 3 days post-injury are indicated by an asterisk (*). Gene trending towards significance but sample expression variability among the control group prevents statistical significance (#)

The overall pattern of gene expression in propriospinal neurons after a local axotomy can be characterized according to their functional family (Table 1). Observed changes in the expression levels of genes categorized as “immune and inflammatory genes” increased after a local axotomy in both the TPS and LDPT populations (Fig. 3a). All four of the genes examined, Fcgr2b, Fyb, Itgam, Lgals3, exhibited a significant increase in the TPS neurons after a T_10_ injury, while only Itgam exhibited a significant increase in expression in the LDPT neurons that were further from the injury site. Not unexpectedly, LDPT neurons that were subject to a local axotomy demonstrated a significant increase in expression in three of the four genes (Fcgr2b, Itgam, and Lgals3) associated with the immune and inflammatory reaction.

Similar changes were observed in the genes related to the cellular process of apoptosis. When the expression of the five genes (Casp2, Casp3, Dap, Gadd45g, and Pycard) (Fig. 3c) were examined, again the TPS neurons exhibited a significant increase in expression in all five genes. In the LDPT neurons that were subjected to a distal axotomy only one gene, the pro-apoptotic gene Pycard, exhibited a significant increase in expression. However, in the LDPT neurons that were subjected to a local axotomy, two genes Dap and Pycard, both associated with being pro-apoptotic, exhibited a significant increase in expression.

Of the growth factor and receptor genes (Fig. 3b), six of the seven genes examined (Bdnf, Gfra1, Igf1, Lifr, Ntrk2, Ncam1 and Ret) exhibited a significant increase in expression in the TPS neurons following a local injury. The only exception was the Ntrk2 gene, which encodes the tyrosine kinase type B receptor. In the LDPT neurons subjected to a distant axotomy, there was little or no change in these genes. Interestingly, the LDPT neurons that experienced a local injury showed little change in these genes either; with the only statistically significant changes being the downregulation of both Lifr, which encodes the receptor for leukemia inhibitory factor, and Ncam1, which encodes the neural cell adhesion molecule 1.

Analysis of the regeneration associated and neuroprotective genes (Fig. 3d), revealed the most interesting findings. This family/grouping of 10 genes included: Actb, Atf-3, Gadd45a, Gap43, Hspb1, Jun, Stat3, Stmn2, Tspo, and Tubb3. As seen with the other family and gene grouping, when it came to the post-injury response of the TPS neurons, all 10 genes in this category demonstrated a significant increase in expression following injury. While the expression levels of two genes, Stat3 and Tspo, increased following a distant injury in LDPT neurons, the response of LDPT neurons subject to a local axotomy was very different. As shown in Fig. 3d, LDPT neurons receiving a local axotomy demonstrated changes for seven of the 10 genes, with significant *increases* in expression being found in the genes Stat3 and Tspo, just as for LDPT neurons with a distant axotomy, and significant *decreases* in expression of the remaining four genes: Gap43, Hspb1, Stmn2, and Tubb3.

The expression of the gene expression regulator gene, Tbp, shows a significant (corrected *p* values < 0.010) increase in expression following a T_10_ level axotomy in TPS neurons, but a non significant decrease (0.90 fold change) in expression in the LDPT neurons that received a distal axotomy. LDPT neurons receiving a local axotomy exhibited a rather meager (1.05 fold increase) in expression post-axotomy.

## Phenotypic differences exist between the TPS and LDPT neuronal populations

One of the most intriguing findings from the current study is the observation that the LDPT neuronal population exhibits a post-axotomy response different than that of the TPS neurons. Moreover, even when the site of axotomy was moved proximally towards the LDPT cell body, the response is different. Axotomy closer to LDPT neurons elicited a dynamic intrinsic post-injury response, but this was not as robust as the changes observed in the TPS neuronal population. The observed differences between the response of LDPT and TPS neurons to a local axotomy invites the question of phenotypic distinction between the TPS and LDPT neuronal populations. The initial study comparing the differential response of LDPT and TPS neurons to axotomy [18] also suggested that they could be distinct cell populations. To further examine this question, the expression levels of the genes specifically tested in this study were compared between the *unaxotomized* TPS and LDPT neurons. Average expression levels of each gene were calculated for the TPS and LDPT neurons harvested from the uninjured control animals (Table 3). Any differences in gene expression levels for the TPS control versus the LDPT control were evaluated for statistical significance.

**Table 3:** Phenotypic differences observed between TPS and LDPT neurons

Gene	LDPT	TPS	Fold change	*P* value	Grouping
Observed phenotypic differences					
*Actb*	*8501.71*	*6179.14*	*0.73*	*0.0421*	RAG
Atf3	9.18	12.08	1.32	0.4635	RAG
Bdnf	20.63	31.57	1.53	0.3192	SRGF
*Casp2*	*4.90*	*12.04*	*2.46*	*0.0032*	AP
Casp3	147.00	90.46	0.62	0.2382	AP
Cybb	1.53	6.05	3.95	0.2659	IM
*Dap*	*29.34*	*66.94*	*2.28*	*0.0159*	AP
*Fcgr2b*	*6.62*	*39.48*	*5.97*	*0.0377*	IM
Fyb	5.58	16.82	3.02	0.1329	IM
*Gadd45a*	*284.22*	*196.00*	*0.69*	*0.0063*	RAG
Gadd45g	5.18	9.66	1.87	0.0616	AP
*Gap43*	*1087.25*	*1300.25*	*1.20*	*0.0413*	RAG
Gfra1	83.58	112.05	1.34	0.1237	SRGF
Hsbp1	1739.43	1436.72	0.83	0.0728	RAG
*Igf1*	*5.51*	*9.68*	*1.76*	*0.0121*	SRGF
*Itgam*	*23.87*	*79.00*	*3.31*	*0.0016*	IM
Jun	119.38	113.87	0.95	0.8782	RAG
Lgals3	13.68	354.43	25.92	0.2606	IM
*Lifr*	*461.73*	*334.60*	*0.72*	*0.0158*	SRGF
Ncam1	854.72	803.53	0.94	0.7075	SRGF
Ntrk2	97.77	94.62	0.97	0.9017	SRGF
*Pycard*	*2.42*	*10.06*	*4.15*	*0.0035*	AP
Ret	444.09	330.41	0.74	0.0754	SRGF
Sox11	1.36	4.22	3.10	0.2533	RAG
Stat3	320.89	327.53	1.02	0.8987	RAG
*Stmn2*	*3837.24*	*2345.47*	*0.61*	*0.0333*	RAG
Tbp	35.70	47.64	1.33	0.1195	RAG
*Tspo*	*5.74*	*37.08*	*6.46*	*0.0023*	RAG
*Tubb3*	*1588.09*	*1060.08*	*0.67*	*0.0353*	RAG

Phenotypic differences between TPS and LDPT Neurons. Phenotypic differences were detected utilizing a *T* test that compared the  baseline expression level of the genes of interest in uninjured LDPT and TPS neurons. Significant *p* values are shown in italics

The “family” that each gene was assigned to is indicated in the far right column *AP* pro/anti apoptotic genes, *IM* immune and inflammatory, *RAG* regeneration associated and/or neuroprotective genes; *SRGF* surface receptor and/or growth factor

One of the first major differences that was found between the two populations was the presence of the pro-apoptotic gene Bax and the inflammatory and immune gene Cxc113 in the TPS, but not LDPT neurons. The expression of these two genes in the TPS neurons corroborates previous findings of their upregulation following injury [17]. Interestingly, expression of Bax or Cxc113 was not observed in the LDPT populations, thus preventing any analyses of these two individual genes. Other notable differences observed between the TPS and the LDPT neuronal populations are summarized in Table 3. Of the genes examined, 13 show significant differences in expression between the TPS and LDPT populations. Ten of these genes show a higher level of expression in the TPS neurons, including all the significant genes in the apoptosis category (Casp2, Dap, Pycard, and Bax) the immune and inflammatory category (Facr2b, Itgam, and Cxc113), two of the regeneration associated genes (Gap43 and Tspo), and one of the surface receptor genes (Igf1). Only five genes are found to be elevated in the LDPT population of neurons. Interestingly, three of those five genes Actb, Stmn2, and Tubb3 are all related to the actin cytoskeleton, and actin cytoskeletal dynamics. Of the remaining two genes, one is a regenerative associated gene (Gadd45a), and the other is the surface receptor gene Lifr.

Comparison of the baseline gene expression in uninjured control LDPT and TPS neurons strongly indicates that phenotypic differences exist between the TPS and LDPT neurons. Moreover, the findings in this current study corroborate the result of a previous study also demonstrating phenotypic differences between TPS and LDPT neurons [18].

## Discussion

This study continues the characterization of the intrinsic post-injury response of PS neurons, and considers the effect of a local axotomy on the response of both LDPT and TPS neuronal populations in the spinal cord. Laser capture microdissection was again utilized to specifically identify and collect Fluorogold retrogradely labeled TPS and LDPT neurons, thus limiting our gene expression profiling to those changes occurring specifically in the TPS or LDPT neurons. However, unlike our previous studies that utilized microarray screening (e.g., Affymetrix rat 1.0 Gene ST array [17, 18]), the present study utilized the Affymetrix Quantigene^®^ Plex 2.0 Assay, to examine the change in gene expression. Ultimately, however, the two methodologies produced similar findings.

Understanding how specific types of neurons respond to injury has become an area of both proteomic and genomic investigation [1, 29–33]. These studies are important to the field of axonal regeneration, because the intrinsic cellular post-injury response and factors that affect it will be critical to stimulating successful axonal regeneration. Perhaps even more important, having a thorough understanding of how individual populations of neurons respond to injury can identify more promising treatments. Specific regenerative components of the post-injury response can be targeted for augmentation in affected cells, while concurrently minimizing any part of the post-injury response that is detrimental to the regenerative process.

Propriospinal neurons have become a neuronal population of interest in the field of spinal cord injury research because of two features: their ability to undergo a dynamic injury-induced neuroplastic reorganization of both spared and injured propriospinal connections [2, 7–12], and their ability to undergo robust regenerative growth after injury [5–7]. The intrinsic post-injury responses of PS neurons appear to contribute to the varying degrees of recovery of function that is observed following trauma to the CNS, where long distance regenerative growth of supraspinal axons fail [1, 2, 10].

Previous work from our lab characterized the post-injury intrinsic response of PS neurons to a T_10_ level axotomy. The interesting finding was that even as both the TPS and LDPT populations of PS neurons are intrinsic to the spinal cord, the post-injury responses exhibited by the TPS and LDPT propriospinal were vastly different [17, 18]. The TPS population mounted a robust post-injury response, which included the upregulation of many regeneration associated, immune and inflammatory, pro/anti apoptotic, and cell surface receptor and growth factor related genes [17]. In contrast, the LDPT population largely remained in a more quiescent state, and even down regulated certain genes related to regeneration, immune and inflammatory pathways, and pro/anti apoptotic genes [18]. These differential responses could be a result of the distance of the injury to the neuronal cell body, and this question was directly addressed in this study.

One potential concern with the design of the study is the use of the retrograde tracer Fluorogold (FG) to pre-label the TPS and LDPT neurons. It has been suggested that FG may exert a cytotoxic effect on neurons over time [34, 35]. However, as reported in previous work [17, 18] no significant changes in gene expression were found comparing control groups at the various post-FG labelling time points (1 week, 2 weeks, or 1 month). Moreover, there was no evidence (genetically or by immunofluorescence) of a pro-apoptotic response in these control groups during the first month following SCI. In particular, the data in this study demonstrate a similar effect in the apoptotic gene family and support the previous findings that FG labeling has no adverse effects on neurons, and is a suitable neuronal tracer for this type of study.

**Fig. 4:**
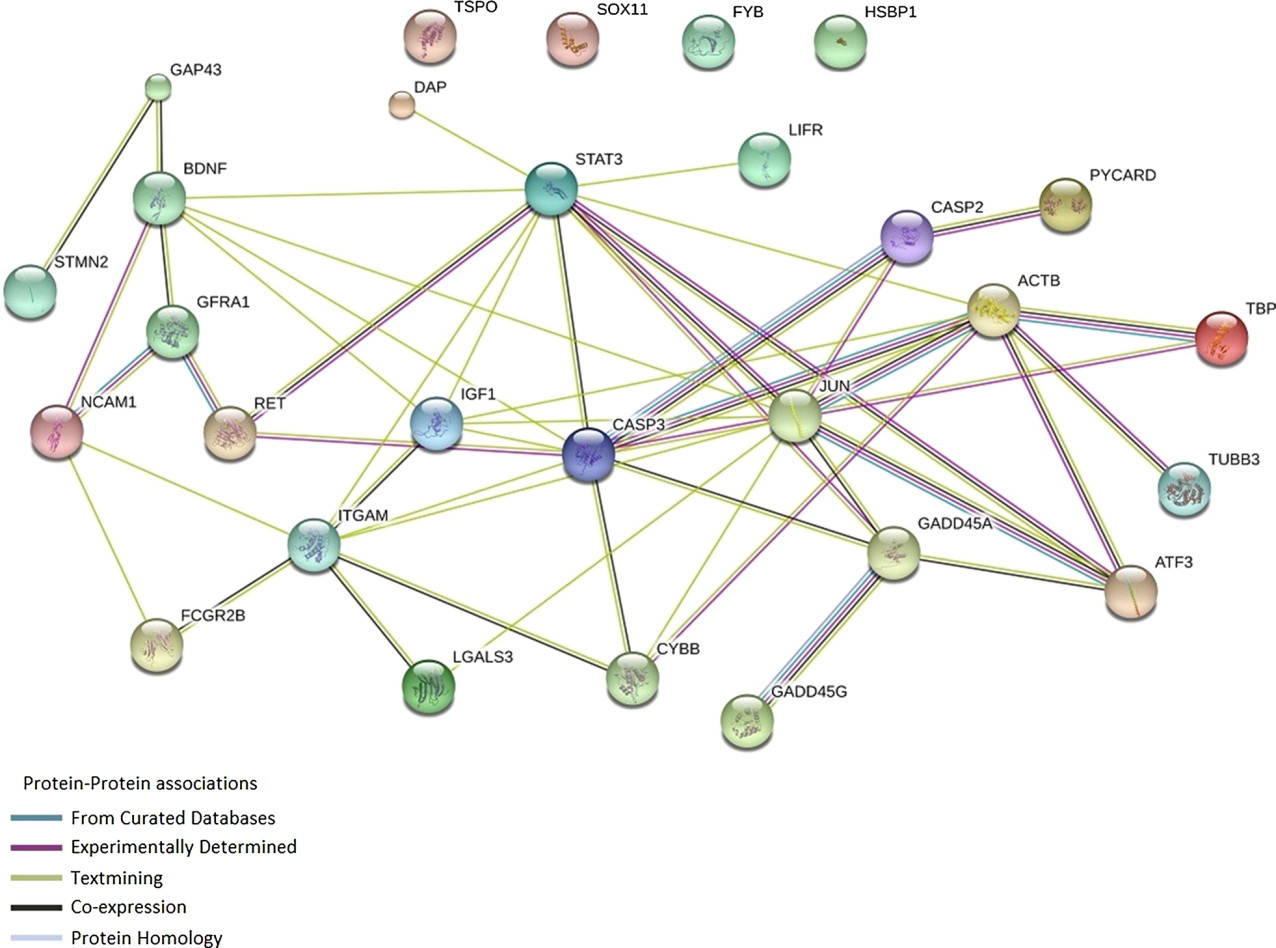
Network Map. The genes selected for this study were uploaded to the online network mapping software STRING, which then establishes the known interactions between the different proteins. As can be seen in the map, only four of the proteins (Hspb1, Tspo, Sox11, and Fyb) are considered orphans with no currently known or observed interactions with all of the other proteins in our study. This network map, visually illustrates which genes interact with which others, and possibly identifies “high value” targets, which could be used to manipulate the post-injury cell response

The selection of genes to profile (Table 1) was based upon our previous studies, and included those found to be significantly up or downregulated 3 days post T_10_ transection in LDPT and TPS neurons following gene microarray, qRT PCR and/or PCR array analyses [17, 18]. Many genes examined in this study span different functional categories (regeneration associated, immune and inflammatory, apoptotic pathways), and we considered that these pathways could potentially interact in the cells. Analysis of gene interactions using the website STRING (https://string-db.org) [36–38], reveals that of the genes examined, only four: Tspo, Sox11, Fyb, and Hspb1 are considered “orphans” with no known or demonstrated direct interactions with the other genes examined (Fig. 4). This demonstrates that most molecules encoded by the genes examined in this study can have some level of interaction with each other, and potentially have a significant influence on the intrinsic response of LDPT neurons. For example, the pro-apoptotic genes Casp2 and Casp3 have a high probability of interaction with each other and other genes in this pathway; similar findings are found with the regenerative associated and neuroprotective genes: Actb, Atf3, Jun, and Stat3, and growth factor and surface receptor genes: Gfra1, and Ret. Interestingly, the gene expression regulator Tbp, has ample evidence of interactions with two of our regeneration associated genes Actb and Jun. This interaction with two known regeneration associated genes, and the fact that all three genes: Tbp, Actb, and Jun, are significantly upregulated in TPS neurons following a T_10_ level lesion, support the idea that they interact. Moreover, the fact that Tbp expression in LDPT neurons is flat, and both Actb and Jun expression is either decreased (as seen in locally injured LDPT neurons) or flat, could indicate that expression of Tbp is a key modulating factor.

Further analysis of the genes of interest using STRING, determining functional enrichments, revealed that the top five biological processes networks highly represented by 13–15 of the genes analyzed were neuronal differentiation, cell development, response to an external stimuli, cell surface receptor signaling, and neurogenesis. Knowing which genes/molecules interact with each other and how they can be influenced by external stimuli will be a critical step in understanding the intrinsic response of individual neuronal populations to environmental changes, including injury. This information could be key to optimizing treatment strategies for injuries or diseases in the nervous system.

## Location of the lesion matters

There is ample evidence that a neuron will mount the strongest regenerative response if the site of axotomy is close to the cell body [5, 6, 25, 26]. In a study conducted by Mason and colleagues, CST neurons were axotomized both intra-cortically and spinally to evaluate the intracellular response. An upregulation of regeneration associated genes classically associated with regeneration (Atf3, Gap43, Chl1, Scg10) was observed in the CST neurons axotomized intra-cortically near the neuronal cell body. These genes were not affected in CST neurons axotomized spinally [26]. A similar effect was observed when RuST neurons (originating in the brain stem) were subjected to either a cervical or thoracic axotomy [25]. The post-injury response of rubrospinal neurons subjected to a cervical axotomy displayed an upregulation in Gap43 and various tubulin proteins that were not observed after thoracic axotomy. The proximity of a spinal lesion to the cell bodies of PS neurons may be one of the reasons why PS axons are able to grow within peripheral nerve grafts, unlike supraspinal neuron axons [5, 6]. In the present study we asked whether axotomizing descending LDPT neurons nearer to their cell bodies, which arise in the cervical enlargement (C_2_–C_7_), would result in a post-injury response and changes in gene expression comparable to the reported changes observed in the TPS neurons following local lesion. We hypothesized that a local axotomy would cause an inflammatory, regenerative, and apoptotic response in LDPT neurons similar to dynamic post-injury response that has been previously reported for TPS neurons [17], and replicated in this present study.

The results from this study clearly demonstrate that moving the axotomy from the T_10_ level to the T_2_ level, has a significant effect on the post-injury response of LDPT neurons. When LDPT neurons are examined after an axotomy at the spinal level of T_10_, the post-injury response is rather meager [18]. Significant changes in gene expression are limited, and many of the observed changes in gene expression actually decrease. However, after an axotomy at spinal level T_2_, nearer to the LDPT cell soma, a different intrinsic post-injury response is observed. There is both a more dynamic post-injury response with a lesion at T_2_, as well as different changes in gene expression, when compared to the response after a distant injury (T_10_ axotomy). After a proximal injury, a series of regeneration and neuroprotective and cell surface receptors genes such as Actb, Gap43, Tubb3, Gfra1 and Ntrk2, are downregulated, compared to the upregulation observed following a T_10_ axotomy. In contrast, the gene for the neurotrophic factor BDNF and anti apoptotic gene Gadd45g, are upregulated in LDPT neurons that received a T_2_ axotomy, compared to their downregulation after a T_10_ axotomy. Other differences in the response of LDPT neurons following a T_2_ level axotomy, included the number of genes exhibiting a significant fold change in expression after a local injury, which was increased three times compared to a distant injury.

Clearly, these data support the hypothesis that the location of the axotomy has a significant effect on the post-injury response of LDPT neurons. The closer to the cell body the axotomy occurs, the more dynamic the post-injury response. In this sense the present study concurs with the studies on supraspinal neuron populations. However, many of the genes involved in a stronger regenerative response in previous studies [5, 6, 25, 26] are down-regulated rather than upregulated in this instance, suggesting that other factors in addition to axotomy location are involved in the LDPT response.

## Propriospinal neurons are not a homogenous population of neurons

While proximity to a spinal lesion had a noticeable effect on gene expression in LDPT neurons, they did not mount the same robust intrinsic response demonstrated as their TPS counterparts. This highlights a key point about the LDPT population; while they are PS neurons, they are phenotypically different from their TPS counterparts.

Previous studies have noted large differences in baseline gene expression between uninjured LDPT and TPS neuronal populations [18]. The current study also compared the normal TPS and LDPT populations and found that of the genes specifically examined, 13 genes exhibited significant differences in baseline expression between the LDPT and TPS neurons (Table 3, [18]). Moreover, Bax, a pro/anti apoptotic gene and Cxcl13, an immune and inflammatory gene were not included in the present analysis because they are *only* expressed in the TPS population. Their lack of expression in LDPT neurons provides evidence that phenotypic differences exist between the LDPT and TPS neuronal populations. It is likely that there are more differences between the LDPT and TPS populations, as this study was limited  in scope.

Of the 13 genes that exhibited a significant difference in baseline expression, five genes: Actb, Gadd45a, Lifr, Stmn2, and Tubb3, exhibited significantly higher expression in LDPT compared to TPS neurons, whereas eight genes: Casp2, Dap, Fcgr2b, Gap43, Igf1, Itgam, Pycard, and Tspo, exhibited significantly lower expression in LDPT neurons when compared to TPS neurons. Actb, Stmn2 (also known as Scg10), and Tubb3 are regeneration associated genes that deal with the actin cytoskeleton [26, 39–41**]**, while Gadd45a is known to be an anti-apoptotic gene and a regeneration associated gene [42]; Lifr is a gene for the surface receptor for the growth factor LIF. There are significant differences in the expression of genes for neurotrophic factor receptors (Cntfr, Gfra1, Gfra2, Lifr, Ntrk1, and Ntrk2), and other genes known to be involved with axonal maintenance (Hspb1, Nf1, Zfp91), which are present at higher levels in LDPT neurons when compared to TPS neurons [18]. These findings appear to suggest that the increased expression of genes involved with axonal maintenance and neurotrophic factors in LDPT neurons may be related to a broader role in axonal function and maintenance.

The genes that had lower expression in LDPT neurons as compared to TPS neurons include three (Casp2, Dap, and Pycard) known to be pro-apoptotic [43–46], and two of the genes, Fcgr2b and Itgam are part of the immune and inflammatory response. The final three genes showing an overall decreased level of expression in LDPT neurons are regeneration associated genes Gap43 and Tspo, and one gene for the growth factor Igf1.

Potential reasons for phenotypic differences between these two populations of PS neurons could be based on their respective anatomy. One of the most obvious differences between LDPT and TPS neurons is the length of their axonal projections. LDPT neurons originate within the intermediate gray matter of the cervical enlargement, and caudally project their axons, terminating within the intermediate gray matter of the lumbosacral enlargement [14, 15]. On the other hand, TPS neurons arise from the thoracic spinal gray matter, and their axons project rostral or caudally for shorter distances [14, 15]. Alternatively, another difference between LDPT and TPS neurons is the number of possible collateral projections [18]. It is hypothesized that the flat post-injury intrinsic response observed in LDPT neurons could possibly be explained if the LDPT neuron is receiving metabolic or other support as the result of “sustaining collaterals”. Such collaterals could interfere with a significant regenerative response, because the neuron is still receiving trophic support. At first sight, the findings from this study would seem to refute the “sustaining collaterals” hypothesis [47, 48], because moving the site of axotomy proximal to the LDPT cell body fails to elicit a robust cellular response similar to what is seen in TPS neurons. The proximal nature of the axotomy should have ‘disconnected’ the LDPT neurons from most (if any) sustaining collateral.

Unfortunately, we still cannot completely rule out or discount the fact that the LDPT population of neurons could have collateral axons arising almost immediately from the origin of the axon, and even have collateral branches that ascend up towards the brain stem [49]. One could still reasonably assume that if PS neurons were a homogeneous population of neurons, then axotomizing the LDPT neurons proximal to their cell body, and removing any possible trophic support from collateral branches that may exist, the intrinsic response of LDPT neurons should mimic that seen in the TPS population. Therefore, the fact that axotomizing LDPT neurons at spinal level T_2_ did not elicit the same response observed in the TPS population, suggests one of two possibilities. First, this LDPT population of neurons has collateral axonal branches arising adjacent to the cell body, and continues to provide neurotrophic support to the LDPT neurons, even after T_2_ axotomy. This explanation is similar to the difference in the post-injury response of RuST neurons to axotomy at upper cervical or thoracic spinal cord [25]. RuST axons send collaterals to both the cervical and lumbar spinal cord, so the collaterals projecting to cervical cord could dampen the regenerative response after thoracic axotomy [25]. Secondly, as described above, there is a fundamental difference in the intrinsic cell biology of these two populations of PS neurons affecting the post-injury response to axotomy.

Further work is needed to develop a complete profile of the phenotypic differences between LDPT and TPS neurons. The findings from this present study corroborate previous findings that reveal LDPT and TPS neurons exhibit phenotypic differences, and that the PS family of neurons is not a homogeneous population. Understanding these differences will be key, if these neurons are to be targeted for therapeutic interventions, because as demonstrated in their intrinsic response to injury, they respond very differently after the same injury.

## Lesion location or inflammation?

It is clear that lesion proximity can have a significant effect on the post-injury response; in this study, the effects are assumed to be attributed to the axonal damage that occurs close to the cell soma. However, another factor that can contribute to the neuronal response is the inflammatory response that also appears quickly in the tissue after a local injury.

The inflammatory response can have mixed effects on axonal regeneration post-SCI. Previous studies have shown the inflammatory response to be detrimental to the reparative process, exacerbating cell loss and the factors that are inhibitory to axonal regeneration [50, 51]. However, another body of literature demonstrates that the inflammatory response may be beneficial for the regenerative response [52, 53]. Certain components of the inflammatory response, *i.e.* the invasion of vascular macrophages, may be needed to stimulate a maximal regenerative response post-axotomy [24, 54–60]. In an experiment conducted by Hossain-Ibrahim and colleagues [59], corticospinal tract axons of adult rats were cut at the C_3_/C_4_ level, and the regenerative response of the CST neurons was studied following the application of the inflammatory agent, lipopolysaccharide (LPS), to the pial surface of the cortex. *In situ* hybridization and immunohistochemical analysis revealed that CST neurons treated with LPS upregulated many classic regeneration associated genes including c-Jun, Atf3, Gap43, and Stmn2 (Scg10). These regeneration associated genes were not upregulated in spinally axotomized CST neurons receiving no LPS treatment. In cases where CST neurons were not axotomized but received LPS treatment, CST neurons upregulated the expression of c-Jun, Atf3, Scg10, and Gap43. This was not observed in the contralateral hemisphere not receiving the LPS. Another example of the beneficial effect of inflammation is described in the study by Lu and Richardson [54], where dorsal root axons were crushed and bacterium *Corynebacterium parvum* was injected into the dorsal root ganglion (DRG). Upon examination, a significant increase in the amount of DRG axonal outgrowth of the dorsal column axons was found, when compared to the controls not exposed to the bacterium [54]. Additionally another inflammatory agent, zymosan has increased the success of dorsal root regeneration following axotomy when applied to DRG neurons [61].

Complementing these findings, retinal ganglion cell axons regenerate most successfully within peripheral nerve grafts and the optic nerve itself with induction of an inflammatory response from a lens injury or other perturbation [55, 57]. Further studies have demonstrated that a specific subclass of vascular macrophage appears to be beneficial to the process of axonal regeneration. Two different subclasses of macrophages have been identified, M1 and M2. In vivo and in vitro experiments indicate that M1 macrophages appeared to be cytotoxic to neurons, while the M2 macrophages actually promoted regeneration, allowing axonal outgrowth across inhibitory chondroitin sulfate proteoglycan barriers [60].

In our earlier work, the TPS neurons, located only two to three spinal segments away from the T_10_ transection location, exhibit a strong upregulation of many immune and inflammatory genes 3 days post-injury [17]. LDPT neurons, whose cell bodies are located many spinal segments rostral to the T_10_ axotomy site, are far away from the injury and not directly exposed to the inflammatory response. Their cellular response is thus affected only by distance. However, when the site of axotomy is moved closer to the cell body (T_2_) the lesion site is now approximately equidistant (2–3 spinal segments) as the T_10_ lesion is to the TPS neuronal cell body. This results in an increase in the expression of immune and inflammatory genes (Fcgr2b, Itgam, and Lgals), which was not seen with a distant injury. The local tissue damage will trigger an inflammatory reaction, which may contribute to the cellular response. Additional studies are needed to further characterize the response to inflammation and the effect of a local axotomy.

## Conclusions

Propriospinal neurons are beginning to garner more attention in the realm of axonal regeneration research because of their robust regenerative and neuroplastic behavior post-injury. This demonstrated neuroplasticity is believed to be partially responsible for some of the observed recovery of function that occurs  after spinal cord injury [2, 8, 10–12]. In order to take maximum advantage of this robust intrinsic neuroplastic response, and possibly drive PS neurons to play a more significant role in the regeneration of the spinal cord following traumatic injury, a comprehensive understanding of the PS intrinsic response to injury is needed.

This study expands on our previous studies [17, 18] characterizing the intrinsic post-injury response of PS neurons, specifically focusing on the TPS and LDPT populations. Utilizing a different methodology to examine the changes in gene expression, this study not only validated the previous findings, but also provided considerable support for the idea that the lesion distance from the cell body has a significant influence on the intrinsic response of the neurons. This finding may help to explain why TPS neurons are involved with the recovery of function that occurs even with the failure of supraspinal axon regeneration. As spinal cord injuries tend to most often occur either in mid thoracic or cervical regions of the spinal cord, injuries at these levels are going to cause an axotomy near the cell body of PS neurons, while the injury is distal to the cell bodies of CST, RuST and other classes of supraspinal neurons.

Another significant finding of the current study was further evidence and confirmation that while both the LDPT and TPS neurons are members of the PS neuron family; PS neurons are not phenotypically homogeneous. Phenotypic differences between LDPT and TPS neurons were found in our previous study [18], and the current study validated that phenotypic differences between TPS and LDPT neurons exist. These differences need to be more thoroughly examined, because as previously discussed, they may help to explain the observed differential intrinsic response between LDPT and TPS neurons.

While further work is needed to more thoroughly understand the post-injury intrinsic response of PS neurons to injury, this study begins to identify certain “keystone” genes that may serve as useful targets for  SCI therapies. Moreover, creating a thorough gene profile of PS neurons should allow us to be able to take maximum advantage of their robust neuroplastic response to injury, allowing for the creation of more regenerative growth and the establishment of functional bypass circuits, allowing for an even greater recovery of function post SCI.

## Methods

All procedures involving the use of animals were approved by the SUNY Upstate Medical University Institutional Animal Care and Use Committee, under the direction of the Department for Laboratory Animal Research, following the provisions and guidelines of the Association for Assessment and Accreditation of Laboratory Animal Care.

Fischer female rats (N = 30, Harlan Labs; East Millstone; NJ) approximately 77 days old (± 10 days) were used in this study. Animals were assigned to various labeling and injury groups as illustrated in Table 4. Data from previous studies demonstrated differences in gene expression within TPS neurons to be maximal 3 days post-injury/axotomy [17], and significant differences in gene expression are already present by this time for LDPT neurons [18]. Therefore, all animals were sacrificed and tissue harvested 3 days post-injury.

**Table 4:** Animal experimental treatment group assignments

Group	N	Label	Condition	Level	Survival post-labeling	Survival post injury
1	10	FG	TXN	T_10_	10 days	3 days
2	10*	FG	TXN	T_2_	10 days	3 days
3	10*	FG	Control	No injury	10 days	–

Animal experimental treatment groups. Female Fischer 344 rats were divided among 3 groups. Using the indicated tracer, Fluorogold (FG) PS neurons were labeled via bilateral injections of FG into the lumbosacral enlargement prior to axotomy. The animals were allowed to recover for 1 week, allowing the FG adequate time to retrogradely label PS neurons. Following 1-week tracer transport time, animal is groups 2 and 3 then received a complete spinal transection (TXN) at the indicated spinal level, and were then allowed to recover for 3-days, at which point the spinal tissue was harvested for laser capture microdissection and gene expression analysis

*Three animals in the T_2_ SCI group and 1 in the uninjured control group were euthanized prior to the planned date due to health complications

## Animal surgeries

### Retrograde labeling of PS neurons

**Fig. 5:**
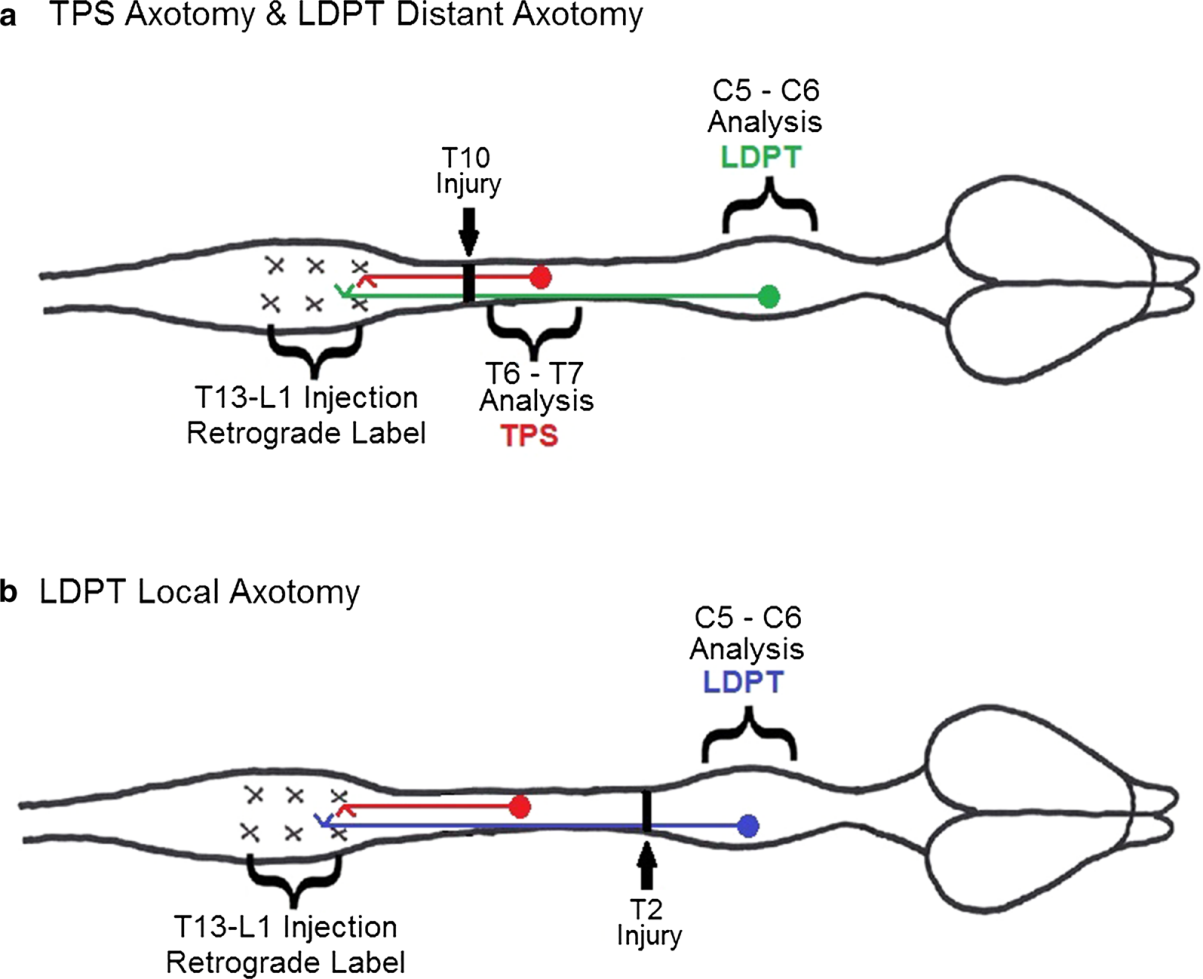
Experimental Schematic. Animals receiving spinal cord injuries were divided into two different injury groups, animals receiving a transection injury at spinal level T_10_ (**a**), and those receiving a transection injury at T_2_ (**b**). All animals received a series of Fluorogold tracer injections (3 bilaterally; approximately 0.3 μL each) in the lumbosacral enlargement. After 1 week tracer transport time, animals were then subjected to a spinal transection at either the T_10_ (**a**) or T_2_ (**b**) spinal level. Following a three day post-injury recovery time, animals were sacrificed, and tissue from the indicated areas was collected for laser capture microdissection

Rats were anesthetized by an intraperitoneal (IP) injection of a ketamine/xylazine cocktail (0.07 cc/100 g). Once the animal was unresponsive to a firm toe pinch, laminectomies were made at the T_13_ vertebral level using aseptic techniques, exposing the rostral aspect of the lumbosacral enlargement. The spinal cord was exposed to open the dura, and a total of six injections (3 bilaterally; approximately 0.3 mL each; Fig. 5) of Fluorogold (FG; Biotinum, Hayward, CA, 3% w/v in dH_2_O) were made using 32G needle attached to a 7901 N 10-mL Hamilton syringe seated in a micrometer injection apparatus. Each injection was performed over an interval of 3–5 min to ensure maximal tracer uptake by the tissue, and following the injection, the needle was left in place for an additional minute to avoid leakage of the tracer from the injection site.

### Spinal transection

Low-thoracic (T_10_) transection injuries were performed as previously described [17, 18]. In brief, rats were anesthetized by an IP injection of a ketamine/xylazine cocktail (100 mg/kg + 10 mg/kg in a volume of 0.07 cc/100 g). Once the animal was areflexic, a laminectomy was made at the T_9_ vertebral level (Fig. 5a) using aseptic technique. The spinal cord was exposed and iridectomy scissors (Fine Science Tools; Foster City, CA) were used to cut the cord (T_10_ spinal level), followed by a probe scraping along the inner wall of the vertebral canal, to ensure a complete transection. Animals in the T_2_ transection injury group were anesthetized, and a laminectomy was performed at the T_2_ vertebral level (Fig. 5b). Once the spinal cord was exposed, the cord was cut using the same procedure as previously described.

### Postoperative care

Following all surgical procedures, the musculature and skin were sutured in anatomical layers. All animals received injections of Cefazolin (30 mg/kg in 0.03 cc SQ) administered twice daily as a prophylactic measure for surgical wound or urinary tract infections. Buprenorphine hydrochloride (Buprenex injectable; Ben Venue Laboratories Inc.; Bedford, OH; 0.1 mg/kg in 0.03 cc SQ) was administered twice daily for the first 48-h for pain management. Spinally injured animals had their bladders manually expressed three times a day for the duration of the study. All animals received additional hydration therapy in the form of lactated Ringer’s solution injections (10 cc SQ), twice daily. All animals had *ad libitum* access to both food and water. Animals in the T_2_ spinal transection group experienced difficulties in feeding themselves, which necessitated enhanced nutritional support in the form of Ensure (Abbott Laboratories).

## Tissue processing and gene expression techniques

### Tissue processing

Following assigned post-injury survival times, animals were euthanized with an IP injection of sodium pentobarbital (Fatal Plus, 150 mg/kg in 0.5 cc), decapitated, and both the mid-thoracic spinal cord (T_5_–T_8_) and cervical enlargement (C_5_–C_7_) promptly dissected out, embedded in O.C.T (Tissue-Tek^**®**^ embedding media; Sakura Finetek USA Inc., Torrance, CA) and rapidly frozen on dry ice. Tissue samples were stored and maintained at − 80 °C until processing. Tissue was sectioned at 20 μm thickness and using a cryostat and mounted on poly-ethylennaphtalae (PEN) foil slides (Leica, Wetzar; Germany). Tissue sections on PEN foil slides were maintained at − 20 °C during the sectioning, and then stored at − 80 °C until laser microdissection (LMD).

### Laser microdissection

Laser microdissection of FG-labeled PS neurons was carried out within a window of 24 h post-sectioning to minimize RNA degradation. Once a slide was removed from − 80 °C, FG-filled neurons were dissected over a 10-min period as described previously [17, 18]. Briefly, slides were positioned on the stage of a Leica AS LMD microscope (Leica Microsystems; Bannockburn, IL). Using a fluorescent filter at 100 ×  magnification retrogradely labeled PS neurons were visualized and individually dissected free of the tissue by manually tracing a laser path around the margins of each neuron of interest. PS neurons were collected from the same region of spinal cord gray matter: intermediate gray matter (laminae V, VII and VIII) and around the central canal (lamina X) of mid-thoracic or cervical enlargement spinal segments. A minimum of 300 FG-labeled neurons were collected from each animal, for both thoracic and cervical levels, from both injured and uninjured control animals. This typically required collection from a total of 20–30 sections per animal.

### RNA purification

**Fig. 6:**
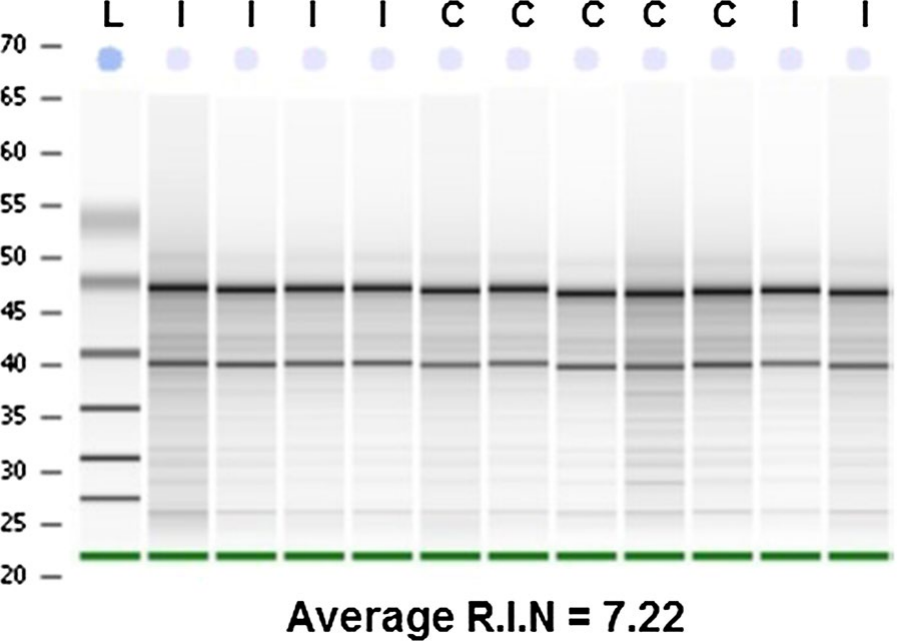
RNA Quality Pseudogel and R.I.N. Fluorogold retrograde labelled neurons were collected by laser capture microdissection, and processed to collect the RNA that was used to measure the changes in genetic expression. The quality of the RNA was assessed using the Qiagen 2100 bioanalyzer (Agilent Technologies; Santa Clara, CA) which provided both an RNA Integrity Number (RIN), and corresponding pseudo gel. L = Ladder, C = Control Animal, and I = Animal receiving spinal transection injury

Laser-dissected neurons were collected directly into a nuclease-free PCR tube cap, containing 30 mL RLT lysis buffer (Qiagen; Valencia, CA) with freshly-added 1% 2-mercaptoethanol (Sigma Aldrich; St. Louis, MO). RNA was purified using the RNeasy Mini kit (Qiagen; Valencia, CA), eluted in 30 mL nuclease-free water and concentrated down to 10 mL by vacuum centrifugation. Total RNA concentration was determined by the RNA 6000 Pico RNA Assay (Agilent Technologies; Santa Clara, CA). Quality of the RNA extraction was determined utilizing a 2100 bioanalyzer (Agilent Technologies; Santa Clara, CA) which provided an RNA Integrity Number (RIN), and corresponding pseudo gel (Fig. 6). The average RIN for the RNA samples in this study was greater than 7.0.

### Selection of genes for analysis

Based upon previous studies, 34 different genes were chosen (Table 1) for quantification at 3 days post-injury for both the T_10_ and T_2_ transection groups. The genes chosen for analysis were found to be significantly up or downregulated 3 days post T_10_ transection in LDPT and TPS neurons following gene microarray, qRT PCR and/or PCR array analyses in our previous studies [17, 18].

### QuantiGene^®^ Plex assay (Affymetrix)

Expression levels for the specific genes of interest were obtained using a highly-sensitive Luminex bead-based assay (Quantigene^®^ Plex 2.0, Affymetrix), as per manufacturer’s instructions (Affymetrix Technical Manual 16659, rev B). Briefly, purified RNA from each sample was hybridized to a mixture of magnetic multi-analyte profiling (xMAP) beads. During this hybridization process, specific mRNA transcripts were captured to their complementary magnetic bead. The double-stranded hybrids were detected and their signals amplified using a branched DNA methodology. The bead identity and signal intensity were read on a Bio-Plex 200 system (BioRad) and the resulting signals analyzed utilizing the Bio-Plex Manager 6.0 software. The limit of detection of each gene analyzed was determined by subtracting the average intensity of the negative control wells from each unknown sample. We subsequently normalized all of the target gene expression values to the internal reference gene Hypoxanthine guanine phosphoribosyltransferase (Hprt).

### Data analysis

Significant increases or decreases in expression were determined by comparing each of the injury groups to their respective uninjured controls. This gene panel was pre-selected from our prior work, and thus more likely to show changes in expression. Data were analyzed using a multifactorial 2-or-3-way Analysis of Variance (ANOVA) incorporating surgical treatment (transection or control), level (thoracic or cervical), and distance from lesion site (proximal or distal) as fixed categorical variables. We also tested if there were significant expression changes in any of the four specific groupings of genes hypothesized to be critically involved in the neuronal response to injury. These groups include genes associated with: regeneration and cell survival/neuroprotection, surface receptor and growth factors, apoptosis, and inflammation (see Table 1).

The significance thresholds for the main effects and interactions between main effects were adjusted using a Benjamini–Hochberg False Discovery Rate (FDR) algorithm to account for multiple testing. When significant main effects or interactions were found, 2-tailed Student’s t-tests were used as post-hoc contrasts to determine the specific conditions that displayed changes in expression. Because our specific genes of interest were chosen based on the findings in our previous published studies, and other work (as described in the Methods: Selection of Genes for analysis), the frequency of our observed changes do not follow any type of random normal distribution. In fact, we observed 82 nominally significant (*p* < .05) and highly correlated test results out of 145 tests, indicating that more than 56% of the genes in our panel were possibly changed. With a preselected gene set, the expected changes show up at a high frequency. In this situation, a more acceptable *p* value (or q value when multiple testing is performed) could be the equivalent of a 1-tailed test rather than 2-tailed test. Utilization of a 0.1 FDR cutoff to determine significance in RNA expression studies, has ample precedent, and has been routinely used in analyses of this type [62–71]. This serves as the basis for our selection of an FDR or q value cutoff < 0.10 in our study.

## Abbreviations

ANOVA: analysis of variance
C_3_: cervical spinal level 3
C_4_: cervical spinal level 4
CNS: central nervous system
CST: corticospinal tract
DRG: dorsal root ganglion
FDR: false discovery rate
FG: fluorogold
IP: intraperitoneal
LAPT: long ascending propriospinal tract
LDPT: long descending propriospinal tract
LPS: lipopolysaccharide
PEN: poly-ethylennaphtalae
PS: propriospinal neurons
RuST: rubrospinal tract
SCI: spinal cord injury
SQ: subcutaneous
T_2_: thoracic spinal level 2
T_10_: thoracic spinal level 10
TPS: short thoracic propriospinal neurons
TXN: spinal transection

## References

[1] Fink KL, Cafferty WBJ (2016). Reorganization of intact descending motor circuits to replace lost connections after injury. Neurotherapeutics.

[2] Isa T (2017). The brain is needed to cure spinal cord injury. Cell Press Rev.

[3] Filous AR, Schwab JM (2018). Determinants of axon growth, plasticity, and regeneration in the context of spinal cord injury. Am J Pathol.

[4] Lang C, Guo X, Kerschensteiner M, Bareyre FM (2012). Single collateral reconstructions reveal distinct phases of corticospinal remodeling after spinal cord injury. PLoS ONE.

[5] David S, Aguayo AJ (1981). Axonal elongation into peripheral nervous system “bridges” after central nervous system injury in adult rats. Science.

[6] Benfy M, Aguayo AJ (1982). Extensive elongation of axons from rat brain into peripheral nerve grafts. Nature.

[7] Courtine G, Song B, Roy RR, Zhong H, Herrmann JE, Ao Y, Qi J, Edgerton VR, Sofroniew MV (2008). Recovery of supraspinal control of stepping via indirect propriospinal relay connections after spinal cord injury. Nat Med.

[8] Flynn JR, Graham BA, Galea MP, Callister RJ (2011). The role of propriospinal interneurons in recovery from spinal cord injury. Neuropharmacology.

[9] Cote MP, Detloff MR, Wade RE, Lemay MA, Houle JD (2012). Plasticity in ascending long propriospinal and descending supraspinal pathways in chronic cervical spinal cord injured rats. Front Physiol.

[10] Filli L, Schwab ME (2015). Structural and functional reorganization of proprispinal connections promotes function recovery after spinal cord injury. Neural Regen Res.

[11] Flynn JR, Conn VL, Boyle KA, Hughes DI, Watanabe M, Velasquez T, Goulding MD, Callister RJ, Grahm BA (2017). Anatomical and molecular properties of long descending propriospinal neurons in mice. Front Neuroanat.

[12] Tohyama T, Kinoshita K, Kobayashi K, Isa K, Watanabe D, Kobayashi K, Lie M, Isa T (2017). Contribution of propriospinal neuron to recovery of hand dexterity after corticospinal tract lesions in monkeys. PNAS.

[13] Chung K, Coggeshall RE (1983). Propriospinal fibers in the rat. J Comp Neurol.

[14] Conta AC, Stelzner DJ (2004). Differential vulnerability of propriospinal tract neuron to spinal cord contusion injury. J Comp Neurol.

[15] Conta AC, Stelzner DJ, Watson C, Paxinos G, Kayalioglu G (2009). The propriospinal system. The spinal cord: a Christopher and Dana Reeve foundation text and atlas.

[16] Stelzner DJ (2008). Short-circuit recovery from spinal injury. Nat Med.

[17] Siebert JR, Middleton FA, Stelzner DJ (2010). Intrinsic response of thoracic propriospinal neurons to axotomy. BMC Neurosci.

[18] Siebert JR, Middleton FA, Stelzner DJ (2010). Long descending cervical propriospinal neurons differ from thoracic propriospinal neuron in response to low thoracic spinal injury. BMC Neurosci.

[19] Conta Steencken AC, Stelzner DJ (2010). Loss of propriospinal neuron after spinal contusion injury as assessed by retrograde labeling. Neuroscience.

[20] Fawcett JW, Asher AA (1999). The glial scar and central nervous system repair. Brain Resch Bull.

[21] Profyris C, Cheema SS, Zang D, Azari M, Boyle K, Petratos S (2004). Degenerative and regenerative mechanisms governing spinal cord injury. Neuro Biol Disease.

[22] Yiu G, He Z (2006). Glial inhibition of CNS axon regeneration. Nat Rev Neuro Sci.

[23] Siebert JR, Conta Steencken A, Osterhout DJ (2014). Chondroitin sulfate proteoglycans in the nervous system: inhibitors to repair. BioMed Res Int.

[24] Richardson PM, Issa VM, Aguayo AJ (1984). Regeneration of long spinal axons in the rat. J Neurocytol.

[25] Fernandes KJL, Fan DP, Tsui BJ, Cassar SL, Tetzlaff W (1999). Influence of the axotomy to cell body distance in rat rubrospinal and spinal motor neurons: differential regulation of Gap-43, Tubulins, and Neurofilament-M. J Comp Neurol.

[26] Mason MRJ, Lieberman AR, Anderson PN (2003). Corticospinal neurons upregulate a range of growth-associated genes following intracortical, but not spinal, axotomy. Eur J Neurosci.

[27] Auble DT (2009). The dynamic personality of TATA binding protein. Trends Biochem Sci.

[28] Ravarani CNJ, Chalanacon G, Breker M, Groot NS, Babu MM (2016). Affinity and competition for TBP are molecular determinants of gene expression noise. Nat Commun.

[29] Xiao H, Huang Q, Zhang F, Bao L, Lu Y, Guo C, Yang L, Huang W, Fu G, Xu S, Cheng X, Yan Q, Zhu Z, Zhange X, Chen Z, Han Z, Zhange X (2002). Identification of gene expression profile of dorsal ganglion in the rat axotomy model of neuropathic pain. PNAS.

[30] Dulin JN, Antunes-Martins A, Chandran V, Costigan M, Lercj JK, Willis DE, Tuszynski MH (2015). Transcriptomic approaches to neural repair. J Nuerosci.

[31] Chandran V, Coppola G, Nawabi H, Tuszynski M, Woolf CJ, Geschwind DH (2016). A systems-level analysis if the peripheral nerve intrinsic axonal growth program. Neuron.

[32] He Z, Jin Y (2016). Intrinsic control of axon regeneration. Neuron.

[33] Cheah M, Fawcett JW, Haenzi B (2017). Differential regenerative ability of sensory and motor neurons. Neurosci Lett.

[34] Naumann T, Härtig W, Frotscher M (2000). Retrograde tracing with Fluoro-Gold: different methods of tracer detection at the ultrastructural level and neurodegenerative changes of back-filled neuron in long-term studies. J Neurosci Methods.

[35] Garret WT, McBride RL, Williams JK, Feringa ER (1991). Fluoro-Gold’s toxicity makes it inferior to True Blue for long-term studies of dorsal root ganglion neurons and motoneurons. Neurosci Lett.

[36] von Mering C, Jensen LJ, Snel B, Hooper SD, Krupp M, Foglierini M, Jouffre N, Huynen MA, Bork P (2005). STRING: known and predicted protein-protein associations, integrated and transferred across organisms. Nucleic Acids Res.

[37] von Mering C, Huynen M, Jaeggi D, Schmidt S, Bork P, Snel B (2003). STRING: a database of predicted functional associations between proteins. Nucleic Acids Res.

[38] Snel B, Lehmann G, Bork P, Huynen MA (2000). STRING: a web-server to retrieve and display the repeatedly occurring neighbourhood of a gene. Nucleic Acids Res..

[39] Lund LM, Machado VM, McQuarrie IG (2002). Increased b-actin and tubulin polymerization in regrowing axons: relationship to the conditioning lesion effect. Exp Neurol.

[40] Avwenagha O, Campbell G, Bird MM (2003). Distribution of GAP-43, β-III tubulin and F-actin in developing and regenerating axons and their growth cones in vitro, following neurotrophin treatment. J Neurocytol.

[41] Mason MR, Lieberman AR, Grenningloh G, Anderson PN (2002). Transcriptional upregulation of SCG10 and CAP-23 is correlated with regeneration of the axons of peripheral and central neuron in Vivo. Mol Cell Neurosci.

[42] Befort K, Karchewski L, Lanoue C, Woolf C (2003). Selective upregulation of the growth arrest DNA damage-inducible gene Gadd45 alpha in sensory and motor neurons after peripheral nerve injury. Eur J Neurosci.

[43] Levy-Strumpf N, Kimchi A (1998). Death associated proteins (DAPs): from gene identification to the analysis of their apoptotic and tumor suppressive functions. Oncogene.

[44] Feinstein E, Druck T, Kastury K, Berissi H, Goodart SA, Overhauser J, Kimchi A, Huebner K (1995). Assignment of DAP1 and DAPK-genes that positively mediate programmed cell death triggered by IFN-g-to Chromosome Regions 5p12.2 and 9q34.1, respectively. Genomics.

[45] Richards N, Schaner P, Diaz A, Stuckey J, Shelden E, Wadhwa A, Gumucio DL (2001). Interaction between Pyrin and the Apoptotic Speck Protein (ASC)Modulates ASC-induced Apoptosis. J Biochem.

[46] Ohtsuka T, Ryu H, Minamishima Y, Macip S, Sagara J, Nakayama KI, Aaronson SA, Lee SW (2004). ASC is a Bax adaptor and regulates the p53-Bax mitochondrial apoptosis pathway. Nat Cell Biol.

[47] Rose JE, Woolsey CN, Harlow HP, Woolsey CN (1958). Cortical connections and functional organization of the thalamic auditory system of the cat. Biological and biochemical bases of behavior.

[48] Fry FJ, Cowan WM (1972). A study of retrograde cell degeneration in the lateral mammillary nucleus of the cat, with special reference to the role of axonal branching in the preservation of the cell. J Comp Neurol.

[49] Grottel K, Krutki P, Mrowczynski W (1999). Bidirectional neurones in the cervical enlargement of the cat spinal cord with axons descending to sacral segments and ascending to the cerebellum and the lateral reticular nucleus. Exp Physiol.

[50] Bareyre FM, Schwab ME (2003). Inflammation, degeneration and regeneration in the injured spinal cord: insights from DNA microarrays. Trends Neurosci.

[51] Fitch MT, Silver J (2008). CNS Injury, glial scars, and inflammation: inhibitory extracellular matrices and regeneration failure. Exp Neurol.

[52] Donnelly DJ, Popovich PG (2008). Inflammation and its role in neuroprotection, axonal regeneration and functional recovery after spinal cord injury. Exp Neurol.

[53] Hauk T, Müller A, Lee J, Schwendener R, Fischer D (2008). Neuroprotective and axon growth promoting effects of intraocular inflammation do not depend on oncomodulin or the presence of large numbers of activated macrophages. Exp Neurol.

[54] Lu X, Richardson PM (1991). Inflammation near the nerve cell body enhances axonal regeneration. J Neurosci.

[55] Leon S, Yin Y, Nguyen J, Irwin N, Benowitz LI (2000). Lens injury stimulates axon regeneration in the mature rat optic nerve. J Neurosci.

[56] Yin Y, Cui Q, Li Y, Irwin N, Fischer D, Harvey AR, Benowitz LI (2003). Macrophage-derived factors stimulate optic nerve regeneration. J Neurosci.

[57] Fischer D, Heiduschka P, Thanos S (2001). Lens-injury-stimulated axonal regeneration throughout the optic pathway of adult rats. Exp Neurol.

[58] Fischer D, He Z, Benowitz LI (2004). Counteracting the Nogo receptor enhances optic nerve regeneration if retinal ganglion cells are in an active growth state. J Neurosci.

[59] Houssain-Ibraham MK, Rezajooi K, MacNally JK, Mason MRJ, Lieberman AR, Anderson PN (2006). Effects of lipopolysaccharide-induced inflammation on expression of growth-associated genes by corticospinal neurons. BMC Neurosci.

[60] Kigerl KA, Gensel JC, Ankeny DP, Alexander JK, Donnelly DJ, Popovich PG (2009). Identification of two distinct macrophage subsets with divergent effects causing either neurotoxicity or regeneration in the injured mouse spinal cord. J Neurosci.

[61] Steinmetz MP, Horn KP, Tom VJ, Miller JH, Busch SA, Mair D, Silver DJ, Silver J (2005). Chronic enhancement of the intrinsic growth capacity of sensory neurons combined with the degradation of inhibitory proteoglycans allows functional regeneration of sensory axons through the dorsal root entry zone in the mammalian spinal cord. J Neurosci.

[62] Zhou Y, Lutz PE, Wang YC, Ragoussis J, Turecki G (2018). Global long non-coding RNA expression in the rostral anterior cingulate cortex of depressed suicides. Transl Psychiatry.

[63] DiTommaso T, Cole JM, Cassereau L, Buggé JA, Hanson JLS, Bridgen DT, Stokes BD, Loughhead SM, Beutel BA, Gilbert JB, Nussbaum K, Sorrentino A, Toggweiler J, Schmidt T, Gyuelveszi G, Bernstein H, Sharei A (2018). Cell engineering with microfluidic squeezing preserves functionality of primary immune cells in vivo. Proc Natl Acad Sci USA.

[64] Yuferov V, Zhang Y, Liang Y, Zhao C, Randesi M, Kreek MJ (2018). Oxygodone Self-administration induces alterations in expression of integrin, semaphoring and ephrin genes in the mouse striatum. Front Psychiatry.

[65] Wang J, Heng YJ, Eliassen AH, Tamimi RM, Hazra A, Carey VJ, Ambrosone CB, de Andrade VP, Brufsky A, Couch FJ, King TA, Modugno F, Vachon CM, Hunter DJ, Beck AH, Hankinson SE (2017). Alcohol consumption and breast tumor gene expression. Breast Cancer Res.

[66] Guan Y, Liang G, Martin GB, Guan LL (2017). Functional changes in mRNA expression and alternative pre-mRNA splicing associated with the effects of nutrition on apoptosis and spermatogenesis in the adult testis. BMC Genom.

[67] Sõber S, Rull K, Reiman M, Ilisson P, Mattila P, Laan M (2016). RNA sequencing of chronic villi from recurrent pregnancy loss patient reveals impaired function of basic nuclear cellular machinery. Sci Rep.

[68] Huan T, Joehanes R, Schurmann C, Schramm K, Pilling LC, Peters MJ, Mägi R, DeMeo D, O’Connor GT, Ferrucci L, Teumer A, Homuth G, Biffar R, Völker U, Herder C, Waldenberger M, Peters A, Zeilinger S, Metspalu A, Hofman A, Uitterlinden AG, Hernandez DG, Singleton AB, Bandinelli S, Munson PJ, Lin H, Benjamin EJ, Esko T, Grabe HJ, Prokisch H, van Meurs JB, Melzer D, Levy D (2016). A whole-blood transcriptome meta-analysis identifies gene expression signatures of cigarette smoking. Hum Mol Genet.

[69] Fang S, Xiong X, Huang L, Chen C (2017). 16S rRNA gene-based association study identified microbial taxa associated with pork intramuscular fat content in feces and cecum lumen. BMC Microbiol.

[70] Ioannidis J, Donadeu FX (2016). Circulating miRNA signatures of early pregnancy in cattle. BMC Genom.

[71] Milani C, Katayama ML, de Lyra EC, Welsh J, Campos LT, Brentani MM, Maciel Mdo S, Roela RA, del Valle PR, Góes JC, Nonogaki S, Tamura RE, Folgueira MA (2013). Transcriptional effects of 1,25 dihydroxyvitamin D(3) physiological and supra-physiological concentration in breast cancer organotypic culture. BMC Cancer.

